# Clinical implications of four different nutritional indexes in patients with IgA nephropathy

**DOI:** 10.3389/fnut.2024.1431910

**Published:** 2024-08-01

**Authors:** Chuyue Qian, Huimin Li, Yue Hou, Wanning Wang, Mindan Sun

**Affiliations:** Department of Nephrology, The First Hospital of Jilin University, Changchun, China

**Keywords:** IgA nephropathy (IgAN), nutrition, index, end-stage renal disease, renal pathology

## Abstract

**Background:**

Immunoglobulin A nephropathy (IgAN) is the most prevalent form of chronic kidney disease (CKD), marked by diverse pathological patterns and variable prognostic outcomes. Nutritional indexes are crucial for disease assessment and prognosis prediction. This study investigates associations between nutritional indexes and renal function in patients with IgAN.

**Methods:**

A cohort of 736 adults diagnosed with IgAN, who underwent renal biopsy at the First Hospital of Jilin University between January 2010 and October 2022, was examined. Clinical and laboratory data were reviewed, and four nutritional indexes were calculated: controlling nutritional status (CONUT) score, geriatric nutritional risk index (GNRI), body mass index (BMI), and prognostic nutritional index (PNI). Cox-proportional hazard analysis evaluated factors associated with end-stage renal disease (ESRD).

**Results:**

Patients with ESRD showed significantly lower GNRI (91.84 vs. 98.94, *p* < 0.001) and median PNI (41.90 vs. 46.30, *p* < 0.001), with higher median CONUT score (2.00 vs. 1.00, *p* = 0.001) compared to those without ESRD. PNI, GNRI, and CONUT scores correlated significantly with C2 in MEST-C classification. Kaplan–Meier analysis indicated increased ESRD probability in individuals with specific thresholds of PNI, GNRI, or CONUT scores. Additionally, GNRI emerged as an independent predictor of ESRD (hazard ratio: 0.963, 95% CI: 0.940–0.979, *p* < 0.001), along with platelet count, serum creatinine, eGFR (CKD-EPI), and triglyceride levels.

**Conclusion:**

GNRI, PNI, and CONUT scores hold potential in reflecting IgAN severity and predicting ESRD risk. GNRI especially may serve as a valuable tool for identifying high-risk individuals for ESRD in IgAN.

## 1 Introduction

IgA nephropathy (IgAN) is the most prevalent form of primary glomerulonephritis globally, with approximately 40% of patients progressing to end-stage renal disease (ESRD) within 10–20 years (1). The hallmark pathology involves the deposition of IgA and complement 3 in the glomerular mesangial region, with increased production and circulation of galactose-deficient IgA1 (gd-IgA1) considered as the initiating factor (2). Furthermore, studies have implicated IgAN in mucosal immune dysfunction and chronic inflammation (3). Managing nutrient intake, particularly protein, is crucial for patients with chronic kidney disease (CKD) to safeguard renal function. However, inadequate dietary intake can compromise immune system function and mucosal integrity (4).

Assessing nutritional status has become a focal point in enhancing disease management, particularly in renal diseases. Despite this, a unanimous standard for nutritional evaluation in renal conditions is lacking. Conventionally, nutritional assessment relies on indicators like body mass index (BMI) or laboratory parameters such as total cholesterol, prealbumin, and albumin levels. However, emerging tools like the controlling nutritional status (CONUT) score, geriatric nutritional risk index (GNRI), and prognostic nutritional index (PNI) have gained traction across various patient demographics, ranging from surgical candidates to individuals with cancer or chronic ailments (5–7). Despite this progress, their potential for predicting the prognosis of IgAN remains largely unexplored. Therefore, this study aims to investigate the correlation between four distinct nutritional indexes and disease severity, along with the likelihood of developing ESRD in patients with IgAN.

## 2 Materials and methods

### 2.1 Study population

We conducted a retrospective review of adults who had undergone renal biopsy at the First Hospital of Jilin University between January 2010 and October 2022 who were diagnosed with IgAN. Out of the initial pool of 1,077 patients, 341 were excluded based on specific criteria: (1) eGFR (CKD-EPI) <15 mL/min/1.73 m^2^ (*n* = 31); (2) presence of autoimmune diseases (*n* = 14); (3) secondary IgAN conditions (*n* = 96), such as hepatitis virus-related glomerulonephritis and Henoch-Schonlein purpura nephritis; (4) acute infectious diseases (*n* = 7) or cancer (*n* = 7); (5) incomplete or absent follow-up data (*n* = 26 and *n* = 160, respectively). Ultimately, 736 eligible patients with IgAN were enrolled in the study. The research protocol underwent thorough scrutiny, was strictly reviewed, and received approval from the ethical committees of the First Hospital of Jilin University (No. 2024-442).

### 2.2 Clinical and laboratory data collection

Following the confirmation of a diagnosis of IgAN through pathology, comprehensive clinical and laboratory data were systematically collected. Clinical parameters included age, gender, mean arterial pressure (MAP), smoking status, alcohol consumption habits, and history of diabetes and/or hematuria. Laboratory measurements included glomerular filtration rate (eGFR), serum albumin levels, blood urea nitrogen (BUN), serum creatinine (Cr), uric acid (UA), total cholesterol (TC), triglycerides (TG), high-density lipoprotein (HDL), low-density lipoprotein (LDL), serum immunoglobulin levels (IgG, IgA, and IgM), complement 3 and 4 levels, C-reactive protein (CRP) levels, anti-streptolysin O (ASO) titers, proteinuria, lymphocyte and leukocyte levels, and platelet counts. eGFR was determined using the CKD Epidemiology Collaboration (CKD-EPI) formula (8). Smoking history was assessed based on the Brinkman Index, calculated as the product of years of smoking and cigarettes smoked per day, with a threshold exceeding 200 indicating significant smoking exposure. Similarly, alcohol history was determined by an index calculated as the product of years of drinking and grams of alcohol consumed per day, with a threshold surpassing 2000 denoting notable alcohol consumption.

### 2.3 Renal pathology evaluation

The Oxford Classification Scoring System (MEST-C) was employed to assess the pathological status of each case. This system classifies cellular/fibrocellular crescents (C), interstitial fibrosis/tubular atrophy (T), segmental glomerulosclerosis (S), endocapillary hypercellularity (E), and mesangial hypercellularity (M) (9).

### 2.4 Selection and calculation of nutritional indexes

Four distinct nutritional indexes were incorporated into our study: PNI, BMI, GNRI, and CONUT score. PNI is calculated as serum albumin (g/L) + 0.005 × blood lymphocyte count (/mm^3^) (10). BMI is determined by weight (kg) divided by height (m) squared. GNRI is computed as (1.519 × serum albumin (g/dL)) + (41.7 × weight (kg)/ideal body weight (kg)), where the ideal weight is calculated as [height (m)]^2^ multiplied by 22 (11). The CONUT score was derived using a formula (Supplementary Table S1) that includes total cholesterol score, lymphocyte count score, and serum albumin score.

### 2.5 Statistical analysis

The statistical analyses were carried out using IBM SPSS Statistics version 24.0. In the analysis results, continuous variables are presented as median with interquartile ranges, while categorical variables are expressed as frequencies and percentages. To compare continuous and categorical variables, appropriate tests, including chi-square or Fisher’s exact test, Kruskal–Wallis test, and the Mann–Whitney U test, were utilized. Spearman’s correlation analysis was also employed to assess the relationships between nutritional indexes and variables.

Receiver operating characteristics (ROC) curve analysis was used to define low and high PNI (or GNRI, CONUT score) with cutoff values set at 42.78, 92.03, or 1.50, respectively. Furthermore, Kaplan–Meier analysis was conducted to compare the renal survival rates of patients with low and high PNI, GNRI, or CONUT score. Additionally, univariable and multivariable Cox-proportional hazard analyses were performed to identify factors associated with ESRD. Prior to conducting multivariable Cox-proportional hazard analysis, variables that had less than a 20% impact on hazard ratios (HRs) of other factors were removed to adjust for confounding variables. A significance level of *p* < 0.05 (two-tailed) was considered statistically significant for all analyses.

## 3 Results

### 3.1 Features of the study population with or without ESRD

This research encompassed 736 patients diagnosed with IgAN. Table 1 illustrates the baseline characteristics of individuals with or without ESRD. The cohort had a median age of 36.00 years, with males comprising 50.14% of the sample. Over a median follow-up period of 26.00 months, 80 patients (10.87%) were identified as having ESRD. Those with ESRD tended to be younger and had higher mean arterial pressure (MAP), along with a notable prevalence of smoking and nephrotic syndrome. Laboratory findings indicated lower hemoglobin levels, platelet count, serum albumin, eGFR, serum IgG, and complement 3 among patients with ESRD, juxtaposed with elevated BUN, Cr, UA, total cholesterol (TC), and proteinuria levels. Renal biopsy data revealed a higher incidence of T1, T2, and C2 lesions in individuals without ESRD. Furthermore, significant disparities in nutritional indexes were evident between the groups, particularly reflected in markedly lower values of prognostic nutritional index (PNI) (median 41.90 vs. 46.30, *p* < 0.001) and geriatric nutritional risk index (GNRI) (median 91.84 vs. 98.94, *p* < 0.001) among patients with ESRD.

**Table 1:** The baseline characteristics of patients with IgA nephritis.

Variables		Total (*n* = 736)	Patients with ESRD (*n* = 80)	Patients without ESRD (*n* = 656)	*p*-value
Clinical characteristic					
Age (years)		36.00 (28.00–46.00)	31.50 (26.00–40.75)	37.00 (29.0–46.00)	0.014
Male, *n* (%)		369 (50.14)	47 (58.75)	322 (49.09)	0.103
Follow-up (months)		26.00 (11.00–53.75)	24.00 (10.00–52.00)	35.00 (16.75–70.75)	0.001
MAP (mmHg)		103.33 (92.33–123.33)	120.17 (97.67–136.67)	102.33 (92.00–121.17)	<0.001
Alcohol, *n* (%)		20 (2.78)	4 (5.00)	16 (2.44)	0.229
Smoking, *n* (%)		71 (9.65)	13 (16.25)	58 (8.84)	0.034
Diabetes, *n* (%)		45 (6.11)	6 (7.50)	39 (5.95)	0.618
Hematuria, *n* (%)		586 (79.62)	66 (82.50)	520 (79.27)	0.498
Nephrotic syndrome, *n* (%)		109 (14.81)	24 (30.00)	85 (12.96)	<0.001
Laboratory data					
Leukocyte count (×10^9^/L)		6.64 (5.67–8.02)	6.57 (5.67–8.07)	6.64 (5.67–8.02)	0.903
Lymphocyte count (×10^9^/L)		2.02 (1.70–2.47)	1.89 (1.53–2.40)	2.04 (1.72–2.48)	0.059
Hemoglobin (g/L)		134.00 (121.00–146.00)	124.00 (114.25–141.00)	135.00 (122.00–147.00)	0.001
Platelet count (×10^9^/L)		242.00 (206.25–291.00)	221.50 (188.25–281.00)	244.00 (207.25–293.75)	0.004
Serum albumin (g/L)		35.80 (32.03–38.90)	33.50 (28.10–36.75)	36.05 (32.30–39.10)	<0.001
BUN (mmol/L)		6.10 (4.81–7.69)	8.56 (6.34–11.29)	5.86 (4.67–7.35)	<0.001
Cr (μmol/L)		90.85 (71.83–122.88)	160.90 (116.25–212.48)	86.95 (69.83–111.83)	<0.001
eGFR (CKD-EPI) (mL/min/1.73 m^2^)		81.10 (56.13–103.30)	42.65 (29.35–60.35)	85.25 (63.53–106.45)	<0.001
UA (mmol/L)		393.00 (320.25–475.00)	441.00 (379.25–532.50)	385.00 (315.00–467.75)	<0.001
TC (mmol/L)		4.99 (4.25–6.00)	5.36 (4.78–6.32)	4.91 (4.21–5.98)	0.011
TG (mmol/L)		1.62 (1.12–2.48)	1.75 (1.13–3.19)	1.61 (1.11–2.45)	0.107
HDL (mmol/L)		1.21 (0.98–1.47)	1.31 (0.99–1.60)	1.20 (0.98–1.45)	0.080
LDL (mmol/L)		3.09 (2.49–3.77)	3.25 (2.51–3.90)	3.08 (2.47–3.75)	0.332
Serum IgG (g/L)		9.43 (7.61–11.30)	7.87 (6.00–10.30)	9.54 (7.74–11.42)	<0.001
Serum IgA (g/L)		2.93 (2.32–3.74)	2.73 (2.18–3.38)	2.95 (2.34–3.79)	0.030
Serum IgM (g/L)		0.96 (0.69–1.30)	0.95 (0.71–1.28)	0.96 (0.69–1.31)	0.978
Complement 3 (g/L)		1.10 (0.97–1.28)	1.02 (0.89–1.16)	1.11 (0.98–1.30)	0.001
Complement 4 (g/L)		0.27 (0.23–0.33)	0.26 (0.22–0.33)	0.27 (0.23–0.33)	0.636
CRP (g/L)		3.02 (1.18–3.23)	3.01 (0.97–3.23)	3.02 (1.28–3.23)	0.306
ASO (U/mL)		55.30 (49.70–100.75)	55.30 (50.08–84.32)	55.30 (49.40–104.00)	0.781
Proteinuria (g/d)		1.94 (1.13–3.66)	3.89 (2.33–6.28)	1.76 (1.07–3.22)	<0.001
Renal biopsy data (Oxford MEST-C), *n* (%)					
M	0	0 (0)	0 (0)	0 (0)	N
	1	736 (100)	80 (100)	656 (100)	
E	0	418 (56.79)	46 (57.50)	372 (56.71)	0.893
	1	318 (43.21)	34 (42.50)	284 (43.29)	
S	0	298 (40.49)	32 (40.00)	266 (40.55)	0.156
	1	438 (59.51)	48 (60.00)	390 (59.45)	
T	0	440 (59.78)	14 (17.50)	426 (64.94)	<0.001
	1	244 (33.15)	43 (53.75)	201 (30.64)	
	2	52 (7.07)	23 (28.75)	29 (4.42)	
C	0	418 (56.79)	34 (42.50)	384 (58.54)	<0.001
	1	234 (31.79)	20 (25.00)	214 (32.62)	
	2	84 (11.41)	26 (32.50)	58 (8.84)	
Nutritional indexes					
PNI		45.85 (41.46–50.19)	41.90 (38.15–47.60)	46.30 (41.91–50.29)	<0.001
BMI		24.22 (21.60–26.98)	23.09 (21.16–25.99)	24.33 (21.78–27.04)	0.024
GNRI		98.43 (89.52–106.58)	91.84 (82.69–102.57)	98.94 (90.99–107.22)	<0.001
COUNT score		2.00 (0.00–3.00)	2.00 (1.00–4.00)	1.00 (0.00–3.00)	0.001

The values are presented as either median (interquartile range) or frequency (percentage). MAP, mean arterial pressure; BUN, blood urea nitrogen; Cr, serum creatinine; eGFR, glomerular filtration rate; UA, uric acid; TC, total cholesterol; TG, triglycerides; HDL, high-density lipoprotein; LDL, low-density lipoprotein; CRP, C-reactive protein; ASO, anti-streptolysin O; PNI, prognostic nutritional index; BMI, body mass index; GNRI, geriatric nutritional risk index; COUNT score, controlling nutritional status score.

### 3.2 Correlation between variables and nutritional indexes

An analysis of the correlation between variables and nutritional indexes was conducted. Notably, nephrotic syndrome, serum albumin, and serum IgG demonstrated significant correlations with PNI, GNRI, and CONUT score, while hemoglobin exhibited a moderate correlation with PNI and GNRI. Proteinuria exhibited the most robust correlation with PNI, with a correlation coefficient of −0.465, indicating a strong negative association. The CONUT score showed a notable correlation with proteinuria, albeit slightly weaker, with a correlation coefficient of 0.379. Importantly, all correlations were statistically significant, with *p*-values less than 0.001. Complement 3 and HDL were moderately correlated with BMI and GNRI. Conversely, while some factors, such as BUN, Cr, eGFR, and UA showed correlations with PNI, BMI, GNRI, or CONUT score, these associations were weak (refer to Table 2).

**Table 2:** The correlation analysis results depicting the relationships between variables and nutritional indexes in IgAN.

Variables	PNI	BMI	GNRI	COUNT
Clinical characteristic				
Age (year)	−0.135 (<0.001)	0.181 (<0.001)	0.037 (0.311)	−0.010 (0.794)
Male	0.171 (<0.001)	0.001 (0.002)	0.171 (<0.001)	−0.077 (0.036)
MAP (mmHg)	0.089 (0.016)	0.251 (<0.001)	0.221 (<0.001)	−0.091 (0.014)
Alcohol	−0.072 (0.050)	0.060 (0.102)	−0.011 (0.765)	0.042 (0.257)
Smoking	0.031 (0.402)	0.042 (0.257)	0.024 (0.523)	−0.060 (0.101)
Diabetes	0.055 (0.139)	0.124 (0.001)	0.107 (0.004)	−0.032 (0.380)
Hematuria	−0.082 (0.027)	−0.120 (0.001)	−0.096 (0.009)	0.038 (0.298)
Nephrotic syndrome	−0.581 (<0.001)	−0.064 (0.084)	−0.540 (<0.001)	0.597 (<0.001)
Laboratory data				
Leukocyte count (×10^9^/L)	0.303 (<0.001)	0.173 (<0.001)	0.166 (<0.001)	−0.191 (<0.001)
Lymphocyte count (×10^9^/L)	0.539 (<0.001)	0.139 (<0.001)	0.193 (<0.001)	−0.321 (<0.001)
Hemoglobin (g/L)	0.358 (<0.001)	0.279 (<0.001)	0.342 (<0.001)	−0.271 (<0.001)
Platelet count (×10^9^/L)	0.073 (0.048)	0.053 (0.152)	0.000 (0.997)	−0.042 (0.255)
Serum albumin (g/L)	0.894 (<0.001)	0.181 (<0.001)	0.794 (<0.001)	−0.761 (<0.001)
BUN (mmol/L)	−0.103 (0.005)	−0.045 (0.220)	−0.090 (0.015)	0.047 (0.200)
Cr (μmol/L)	−0.036 (0.331)	0.038 (0.304)	0.026 (0.483)	0.048 (0.193)
eGFR (CKD-EPI) (mL/min/1.73 m^2^)	0.131 (<0.001)	−0.053 (0.154)	0.021 (0.561)	−0.083 (0.024)
UA (mmol/L)	0.146 (<0.001)	0.187 (<0.001)	0.214 (<0.001)	−0.109 (0.003)
TC (mmol/L)	−0.279 (<0.001)	0.080 (0.029)	−0.237 (<0.001)	0.014 (0.703)
TG (mmol/L)	0.068 (0.063)	0.442 (<0.001)	0.244 (<0.001)	−0.112 (0.002)
HDL (mmol/L)	−0.289 (<0.001)	−0.327 (<0.001)	−0.454 (<0.001)	0.187 (<0.001)
LDL (mmol/L)	−0.246 (<0.001)	0.119 (0.001)	−0.187 (<0.001)	0.005 (0.901)
Serum IgG (g/L)	0.437 (<0.001)	0.017 (0.647)	0.385 (<0.001)	−0.402 (<0.001)
Serum IgA (g/L)	0.150 (<0.001)	0.079 (0.032)	0.174 (<0.001)	−0.128 (<0.001)
Serum IgM (g/L)	−0.094 (0.011)	−0.185 (<0.001)	−0.153 (<0.001)	0.058 (0.113)
Complement 3 (g/L)	0.189 (<0.001)	0.406 (<0.001)	0.338 (<0.001)	−0.182 (<0.001)
Complement 4 (g/L)	0.003 (0.927)	0.271 (<0.001)	0.156 (<0.001)	−0.041 (0.271)
CRP (g/L)	0.041 (0.261)	0.192 (<0.001)	0.159 (<0.001)	−0.025 (0.503)
ASO (U/mL)	0.126 (0.001)	−0.077 (0.036)	0.051 (0.166)	−0.090 (0.015)
Proteinuria (g/d)	−0.456 (<0.001)	0.140 (<0.001)	−0.135 (<0.001)	0.379 (<0.001)

MAP, mean arterial pressure; BUN, blood urea nitrogen; Cr, serum creatinine; eGFR, glomerular filtration rate; UA, uric acid; TC, total cholesterol; TG, triglycerides; HDL, high-density lipoprotein; LDL, low-density lipoprotein; CRP, C-reactive protein; ASO, anti-streptolysin O.

**Figure 1:**
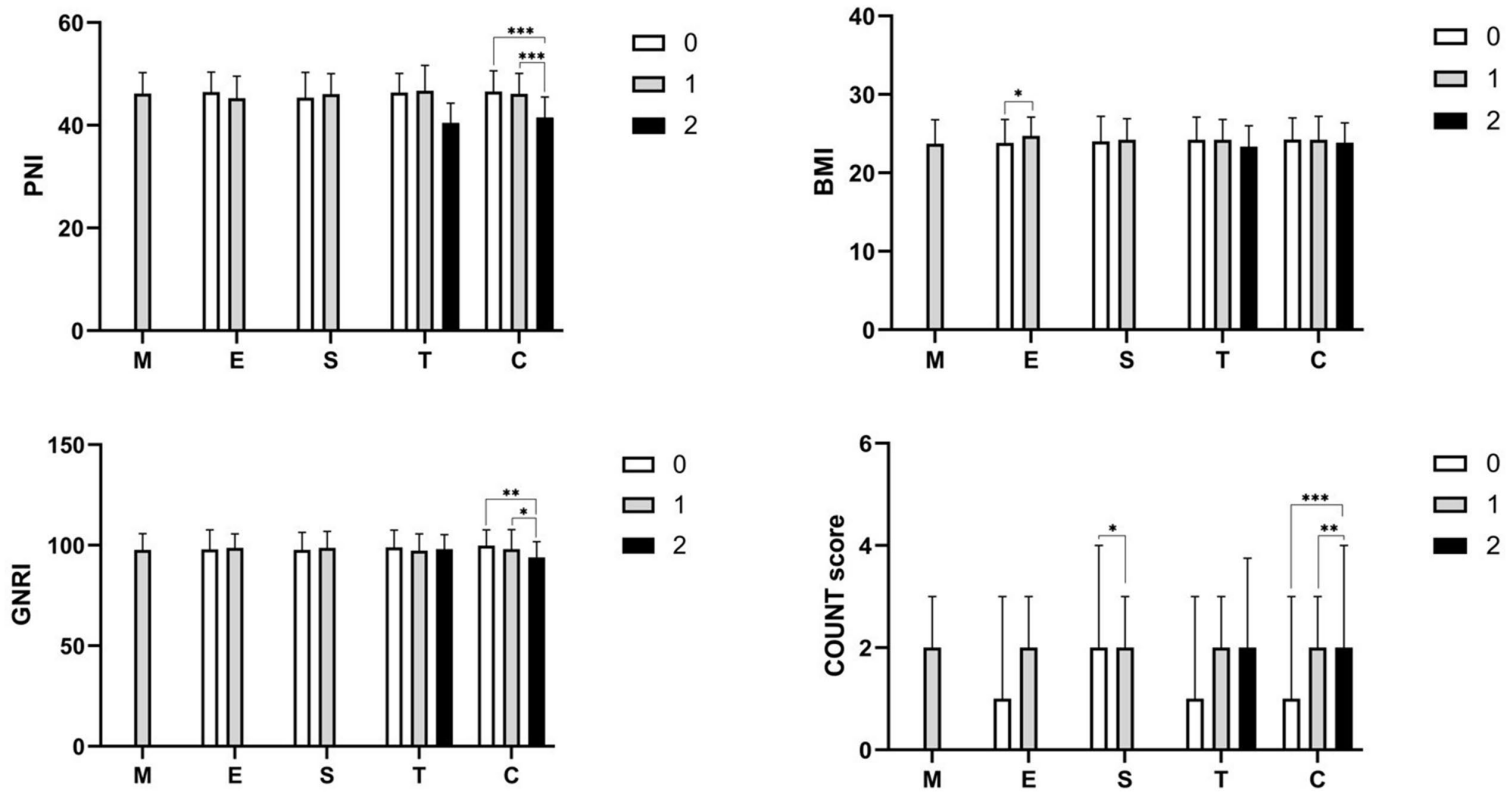
The comparison of various nutritional indexes categorized by MEST-C classification. Statistical significance levels are denoted as *p < 0.05, **p < 0.01, and ***p < 0.001 between the different groups.

Moreover, the study compared four nutritional indexes across the MEST-C classification. PNI, GNRI, and CONUT score demonstrated similar trends concerning cellular/fibrocellular crescents (C) classification. Specifically, patients classified as C2 exhibited lower PNI and GNRI scores compared to those in C0 and C1, whereas the CONUT score showed the opposite trend (refer to Figure 1). No significant differences were observed in other groups.

### 3.3 Factors associated with ESRD in patients with IgAN

**Figure 2:**
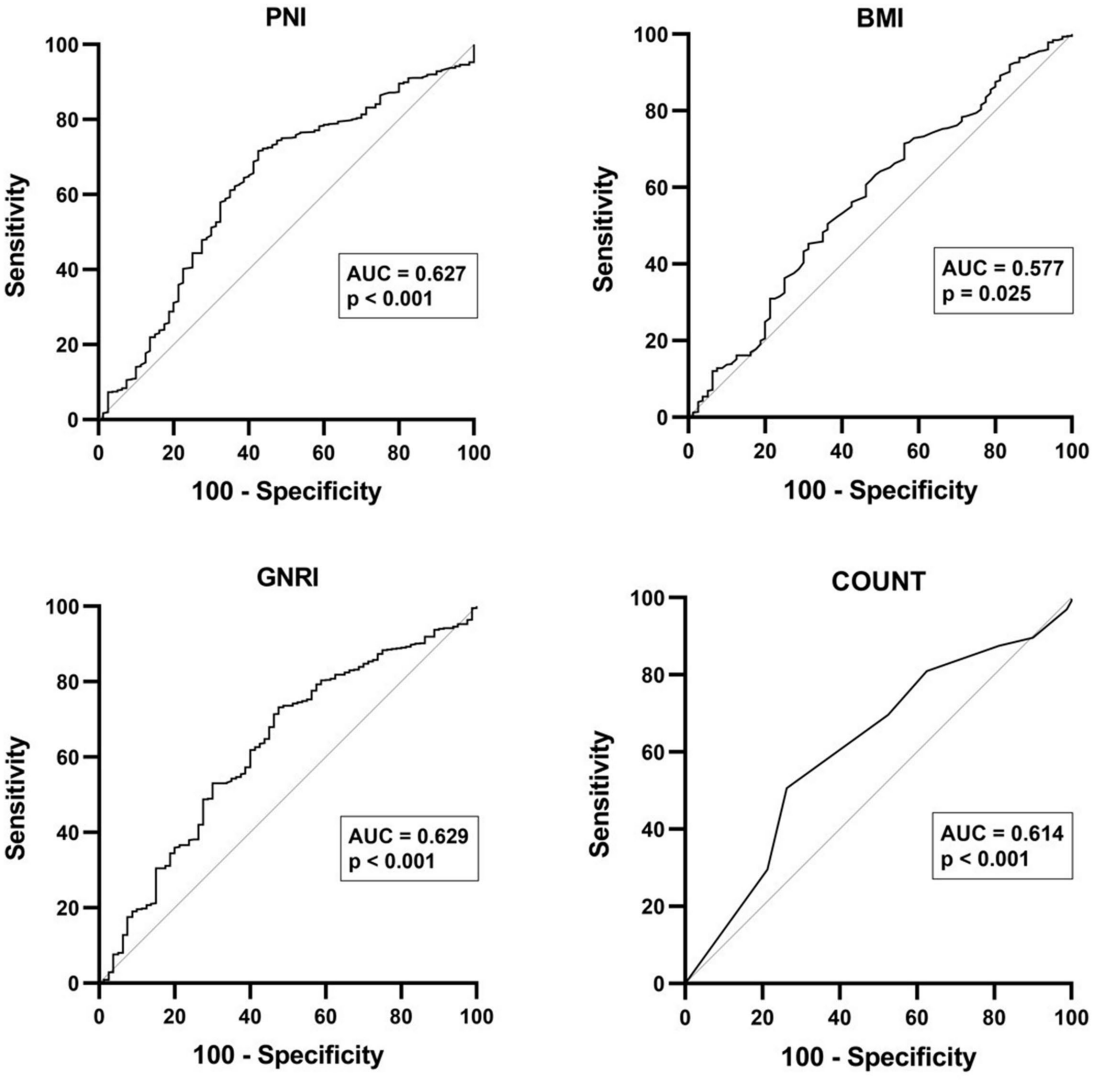
The receiver operating characteristic (ROC) curve analysis for PNI, BMI, GNRI, and CONUT score in predicting ESRD among patients with IgA nephritis.

Among the four nutritional indexes analyzed, PNI (AUC = 0.627, sensitivity 71.6%, specificity 57.5, 95% CI 0.560–0.693, *p* < 0.001), GNRI (AUC = 0.629, sensitivity 73.2%, specificity 52.5, 95% CI 0.563–0.694, *p* < 0.001), and CONUT (AUC = 0.614, sensitivity 73.8%, specificity 50.6, 95% CI 0.320–0.452, *p* < 0.001) demonstrated potential in predicting ESRD. Figure 2 depicts optimal cutoff values for predicting ESRD in the analysis provided, which were determined to be PNI ≤ 42.78, GNRI ≤ 92.03, and CONUT ≥ 1.50. These values were identified as key thresholds indicating heightened risk of ESRD development.

**Figure 3:**
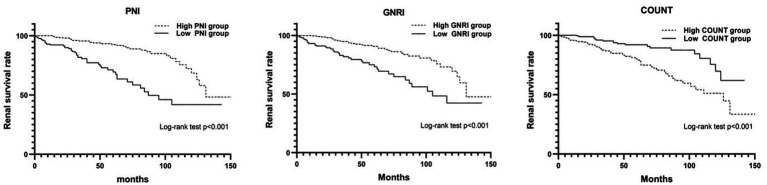
The Kaplan–Meier curve analysis for depicting renal survival rate categorized by PNI, GNRI, and COUNT score in IgAN.

Subsequently, a Cox-proportional hazard analysis was conducted to identify factors associated with ESRD. Various factors used in the univariable analysis, including nephrotic syndrome, mean arterial pressure (MAP), hemoglobin, platelet count, serum albumin, BUN, Cr, eGFR, UA, TG, serum IgG, serum IgA, complement 3, proteinuria, E1, T1, T2, C1, C2, PNI, GNRI, and CONUT were significantly associated with ESRD. The Kaplan–Meier analysis showed that individuals with PNI ≤ 42.78, GNRI ≤ 92.03, or CONUT ≥ 1.50 exhibited a substantially elevated probability of progressing to ESRD, as illustrated in Figure 3. These specific cutoff values served as important indicators of increased risk for ESRD onset. However, in multivariable analysis, only GNRI, platelet count, Cr, eGFR, and TG emerged as independent factors for ESRD (refer to Table 3). In the multivariable analysis includes GNRI and clinical parameters (total cholesterol, platelet count and glomerular filtration rate), the concordance index (C-index) (0.924, 95%CI 0.899–0.949) shows a good aggregate concordance, which is the highest among the multivariable analysis of four nutritional indexes with the same clinical parameters (Supplementary Table S2).

**Table 3:** The results of Cox-proportional hazard analysis.

Variables	Univariable analysis		Multivariable analysis	
	HR (95% CI)	*p*-value	HR (95% CI)	*p*-value
MAP (mmHg)	1.021 (1.013–1.030)	<0.001		
Nephrotic syndrome	3.106 (1.922–5.020)	<0.001		
Hemoglobin (g/L)	0.976 (0.965–0.987)	<0.001		
Platelet count (×10^9^/L)	0.994 (0.990–0.998)	0.007	0.994 (0.990–0.998)	0.005
Serum albumin (g/L)	0.951 (0.925–0.978)	<0.001		
BUN (mmol/L)	1.371 (1.297–1.450)	<0.001		
Cr (μmol/L)	1.024 (1.020–1.027)	<0.001	1.016 (1.010–1.023)	<0.001
eGFR (CKD-EPI) (mL/min/1.73 m^2^)	0.942 (0.932–0.953)	<0.001	0.977 (0.962–0.993)	0.005
UA (mmol/L)	1.005 (1.003–1.007)	<0.001		
TG (mmol/L)	1.077 (0.979–1.186)	0.003	1.242 (1.099–1.404)	0.001
Serum IgG (g/L)	0.851 (0.790–0.917)	<0.001		
Serum IgA (g/L)	0.716 (0.566–0.906)	0.005		
Complement 3 (g/L)	0.325 (0.123–0.858)	0.023		
Ptoteinuria (g/d)	1.165 (1.108–1.226)	<0.001		
E1	1.824 (1.158–2.873)	0.009		
T1	4.574 (2.496–8.383)	<0.001		
T2	11.787 (6.040–23.002)	<0.001		
C1	1.020 (0.587–1.775)	0.943		
C2	5.208 (3.100–8.751)	<0.001		
PNI	0.953 (0.929–0.977)	<0.001		
GNRI	0.973 (0.958–0.988)	<0.001	0.963 (0.946–0.979)	<0.001
COUNT score	1.194 (1.085–1.314)	<0.001		

MAP, mean arterial pressure; BUN, blood urea nitrogen; Cr, serum creatinine; eGFR, glomerular filtration rate; UA, uric acid; TG, triglycerides; PNI, prognostic nutritional index; GNRI, geriatric nutritional risk index; COUNT score, controlling nutritional status score.

## 4 Discussion

This study represents the inaugural assessment of the clinical utility of four distinct nutritional indexes in patients diagnosed with IgAN. Among these indexes, the PNI, GNRI, and CONUT score demonstrated superior correlations with the severity of crescents in patients with IgAN and were also linked with the risk of progressing to ESRD. Notably, both the PNI and GNRI were significantly lower in patients with IgAN who developed ESRD. However, in the multivariable Cox-proportional hazard analysis, only GNRI emerged as a predictor of ESRD, alongside platelet count, serum creatinine, and eGFR calculated using the CKD-EPI equation, and triglyceride levels.

The presence of cellular or fibrocellular crescents in patients with IgAN has been associated with adverse renal outcomes (12). Specifically, crescents present in over 25% of glomeruli (classified as C2 in MEST-C) independently predicted the development of ESRD, even in patients undergoing corticosteroid or immunosuppressive therapy (12–14). Recent investigations have indicated that GNRI serves as an independent marker reflecting the extent of crescentic lesions, suggesting its potential utility in monitoring crescent severity (15). Our study further revealed that not only GNRI but also PNI and CONUT score exhibited strong correlations with crescent proportions.

Furthermore, both PNI and CONUT score exhibited significant associations with proteinuria levels, serum IgG, and serum albumin. Serum albumin, serving as a gauge of nutritional status and an acute-phase protein, is reduced during systemic inflammatory responses and has been recognized as an independent risk factor for ESRD in IgAN (15). Elevated serum IgG levels have been correlated with heightened crescentic lesions and improved cumulative renal survival rates (16, 17). Proteinuria, a hallmark characteristic of IgAN, not only signifies disease activity (where increased proteinuria can precipitate hypoalbuminemia) but also mirrors systemic inflammation and endothelial dysfunction (18).

Furthermore, decreased levels of serum complement 3, indicative of systemic inflammation, were noted in patients with IgAN and ESRD. A lower median complement 3 (1.02 vs. 1.11, *p* = 0.001) demonstrated a notable correlation with BMI and Geriatric Nutritional Risk Index (GNRI) (*r* = 0.406 and *r* = 0.338, respectively; all *p* < 0.001). Intense mesangial complement 3 deposition was linked to more severe pathological lesions, underscoring the pivotal role of complement 3 in the progression of IgAN (19). Furthermore, low serum complement 3 levels and mesangial complement 3 deposition were identified as independent risk factors for renal progression in IgAN (20).

GNRI transcends its conventional role as a mere nutritional assessment tool. Recent research suggests its potential association with inflammation indicators in elderly patients and systemic inflammatory response in cancer cases (21, 22). Within the context of IgAN, our study delves into the correlation between GNRI and serum markers such as albumin, IgG, and complement 3, illuminating the intricate interplay between nutrition and inflammation (23). As evidence mounts regarding their intertwined nature, the pressing question arises: how do we tailor nutritional interventions for patients with IgAN?

Nephrologists have long recognized the pivotal role of dietary adjustments in slowing CKD progression, offering clinicians standardized guidelines. While moderating protein intake remains central to alleviating renal strain, studies underscore the peril of insufficient nutrition, culminating in immune system dysregulation and gut mucosal impairment (24).

Recent insights spotlight the gut’s pivotal role in IgAN pathogenesis (25), underscoring the intricate interplay between gut-associated lymphoid tissue and renal health, dubbed the gut-kidney axis. The advent of targeted interventions like Nefecon, designed to modulate gut mucosal function, underscores this nexus. Hence, there is a palpable need for updated dietary guidelines tailored to patients with IgAN, particularly those grappling with weight management or stringent dietary restrictions.

Recent strides in IgAN therapeutics, exemplified by Nefecon, an oral formulation of budesonide tailored to target the gut mucosa, has emerged as a groundbreaking therapy for IgAN. Through its efficacy demonstrated in the first phase 3 trial, marked improvements in key indicators such as the 24 h urine protein-to-creatinine ratio (UPCR) and estimated glomerular filtration rate have been observed (26). This underscores the pivotal role of the gut in IgAN pathogenesis, reaffirming the concept of the gut-kidney axis.

Given these advancements, there is a compelling case for revisiting dietary guidelines for patients with IgAN to better manage renal burden and disease progression. This is especially pertinent for individuals grappling with weight management or facing stringent dietary constraints. For instance, adopting a gluten-free diet presents a promising approach. Studies have highlighted heightened gut permeability in IgAN patients (27), with gluten potentially exacerbating this phenomenon by triggering the production of IgA anti-alimentary antigens (28).

Compelling evidence further links celiac disease with an increased likelihood of IgAN development (29), underscoring the potential impact of dietary interventions. Animal studies have provided insights into the mechanisms at play, with findings suggesting a correlation between gluten-rich diets and heightened renal IgA deposition, indicative of experimental IgAN induction (30). However, the absence of randomized controlled trials exploring the efficacy of gluten-free diets in IgAN necessitates further research in this domain.

### 4.1 Study limitations and future avenues

This retrospective study has limitations, including inherent biases stemming from selection biases and missing data. Additionally, the scarcity of patients with IgAN and M0 lesions in our biopsy cohort poses a challenge, precluding their inclusion in the study.

Moving forward, two avenues merit further exploration: firstly, stratifying GNRI to identify optimal thresholds, which is crucial given the prevalence of malnutrition coexisting with obesity in patients with CKD (31), thereby necessitating nuanced nutritional assessments. Among a cohort exceeding 3 million US veterans, an intriguing pattern emerged in the association between BMI and kidney function decline, revealing a U-shaped relationship. This trend was particularly pronounced with advancing age (32), suggesting a nuanced interplay between BMI and renal health among the elderly. Meanwhile, GNRI, comprising parameters like albumin, height, and weight, warrants careful consideration. Notably, excessive weight can skew GNRI readings, potentially yielding misleading results. Hence, ensuring accurate interpretation of GNRI necessitates accounting for factors such as body composition and underlying health conditions to avoid erroneous assessments.

Secondly, integrating GNRI with lymphocyte count, which is common in PNI and COUNT score, given its previously established association with crescentic lesions in IgAN, holds promise for refining disease severity indexes.

## 5 Conclusion

Our findings underscore the utility of GNRI, PNI, and COUNT score as markers reflecting crescentic proportions in patients with IgAN, with implications for ESRD risk stratification. Notably, GNRI emerges as a potentially valuable tool for identifying high-risk patients with IgAN warranting closer monitoring for ESRD progression.

## Data availability statement

The raw data supporting the conclusions of this article will be made available by the authors, without undue reservation.

## Ethics statement

The studies involving humans were approved by the Ethics Committee of the First Hospital of Jilin University. The studies were conducted in accordance with the local legislation and institutional requirements. The ethics committee/institutional review board waived the requirement of written informed consent for participation from the participants or the participants’ legal guardians/next of kin because the data being analyzed in our retrospective research is completely anonymized. There is no direct involvement or impact on the individuals whose data is being studied. Obtaining informed consent from each participant would be impractical and unnecessary, as the research does not pose any risks or potential harm to the subjects.

## Author contributions

CQ: Conceptualization, Data curation, Formal analysis, Methodology, Software, Writing – original draft, Writing – review & editing. HL: Data curation, Methodology, Software, Writing – review & editing. YH: Formal analysis, Validation, Writing – review & editing. WW: Supervision, Validation, Writing – review & editing. MS: Supervision, Writing – review & editing.
