# Supplementary material for: Clinical implications of four different nutritional indexes in patients with IgA nephropathy
Source: Front Nutr. 2024 Aug 1;11:1431910. doi: 10.3389/fnut.2024.1431910 (PMC11324556; doi:10.3389/fnut.2024.1431910)
Supplement: Supplementary file 1 [file Table_1.docx]

Supplementary Material

# Supplementary Table1. Calculation of the CONUT score.

| CONUT score  (0-12) | Elements |  | Scores |
| --- | --- | --- | --- |
|  | Serum albumin (g/L) | ≥35 | 0 |
|  |  | 30-34 | 2 |
|  |  | 25-29 | 4 |
|  |  | <25 | 6 |
|  | Total cholesterol (mg/dl) | >180 | 0 |
|  |  | 140-180 | 1 |
|  |  | 100-139 | 2 |
|  |  | <100 | 3 |
|  | Total lymphocytes (×10^9^/L) | >1.6 | 0 |
|  |  | 1.2-1.6 | 1 |
|  |  | 0.8-1.199 | 2 |
|  |  | <0.8 | 3 |

# Supplementary Table2. Concordance index of the Cox-proportional hazard analysis with four nutritional indexes. The multivariable analysis includes total cholesterol, platelet count and glomerular filtration rate.

|  | **Univariable Analysis** | | **Multivariable Analysis** | |
| --- | --- | --- | --- | --- |
| **Variables** | **concordance index** | **95% CI** | **concordance index** | **95% CI** |
| BMI | 0.560 | 0.482-0.638 | 0.917 | 0.890-0.944 |
| PNI | 0.717 | 0.660-0.774 | 0.921 | 0.896-0.946 |
| GNRI | 0.673 | 0.606-0.740 | 0.924 | 0.899-0.949 |
| COUNT score | 0.670 | 0.603-0.737 | 0.919 | 0.894-0.944 |

BMI: body mass index; GNRI: geriatric nutritional risk index; COUNT score: controlling nutritional status score.
